# Prognostic Factors and Imaging Strategies in Unknown Subarachnoid Hemorrhage: A Retrospective Study

**DOI:** 10.7759/cureus.83352

**Published:** 2025-05-02

**Authors:** Monami Dai, Kimura Tatsuki, Shunsuke Ikeda, Taro Yanagawa, Shinichiro Yoshikawa, Tsuyoshi Uesugi, Toshiki Ikeda

**Affiliations:** 1 Stroke Center, Sagamihara Kyodo Hospital, Sagamihara, JPN

**Keywords:** angiography, diagnosis, dsa, sah, unknown sah

## Abstract

Background

Subarachnoid hemorrhage (SAH) without an identifiable vascular lesion on initial angiography, commonly referred to as unknown or angiogram-negative SAH, remains diagnostically and prognostically challenging. Although perimesencephalic patterns tend to be benign, diffuse hemorrhages are associated with worse outcomes. The role of repeat vascular imaging in improving diagnosis and predicting prognosis remains unclear.

Objective

This study aimed to identify clinical and radiological factors associated with outcomes in patients with unknown SAH and evaluate the diagnostic and prognostic value of repeat angiography.

Methods

We retrospectively analyzed 26 patients with spontaneous, non-traumatic SAH and negative findings on initial digital subtraction angiography (DSA). Clinical status, hemorrhage patterns, and imaging data were recorded. Outcomes were assessed at discharge using the modified Rankin Scale (mRS). Repeat angiographic modalities and timing were analyzed in relation to prognosis.

Results

Patients with focal hemorrhage patterns, higher Glasgow Coma Scale (GCS) scores at admission, and absence of hydrocephalus had significantly better outcomes. Repeat DSA was more frequently performed in the good outcome group, although no new vascular lesions were identified.

Conclusion

Unknown SAH is generally associated with favorable outcomes, but vigilance is warranted in diffuse SAH cases. Repeat DSA may not always reveal new lesions, but it plays a valuable role in guiding clinical confidence and management decisions. Tailored imaging strategies based on clinical and radiological risk factors are recommended.

## Introduction

Subarachnoid hemorrhage (SAH) without an identifiable vascular source on initial angiography, termed angiogram-negative or unknown SAH, accounts for 10-15% of spontaneous, non-traumatic SAH cases. Several possible etiologies have been proposed for these cases, including thrombosed aneurysms, ruptured perforating arteries, venous bleeding, or microvascular malformations that are undetectable by routine imaging [1,2].

Perimesencephalic SAH is typically defined as a localized hemorrhage centered anterior to the midbrain and pons, without significant extension into the Sylvian fissures, ventricles, or cortical sulci. It is often considered a benign subtype of SAH, generally associated with favorable outcomes. In contrast, diffuse SAH involves more widespread bleeding, often extending into multiple cisterns or the ventricular system. This broader distribution of blood may increase the risk of complications by promoting inflammatory responses, impairing cerebrospinal fluid circulation, and irritating cerebral vessels, thereby leading to hydrocephalus and vasospasm [2,3]. 

Digital subtraction angiography (DSA) remains the gold standard for vascular evaluation, yet a single negative DSA may miss subtle lesions due to vasospasm, clot obstruction, or technical limitations [4,5]. While repeat imaging, especially second and third DSAs, can detect previously hidden vascular pathologies, the timing, frequency, and modality of such imaging remain controversial [6,7].

This study aimed to investigate the clinical and radiological predictors of short-term functional outcomes in patients with angiogram-negative SAH. In particular, we evaluated the diagnostic contribution of repeat vascular imaging (DSA, computed tomography angiography (CTA), or magnetic resonance angiography (MRA)) and explored the implications of hemorrhage distribution patterns, including perimesencephalic and diffuse SAH. We further aimed to clarify whether repeat angiography influences outcome or decision-making in the management of these patients.

## Materials and methods

This retrospective study included 26 patients who were diagnosed with spontaneous, non-traumatic SAH and demonstrated no vascular lesions on initial DSA. All patients were admitted to Sagamihara Kyodo Hospital located in Sagamihara, Kanagawa, Japan, between 2021 and 2024. Exclusion criteria included trauma-related hemorrhage, coagulopathy, or known cerebrovascular malformations. Clinical data collected included age, sex, and neurological status at admission, assessed using the Glasgow Coma Scale (GCS), World Federation of Neurosurgical Societies (WFNS) grade, Fisher grade, and Hunt and Kosnik (H&K) grade. The hemorrhage pattern was classified as focal or diffuse based on initial imaging. We also assessed the presence of hydrocephalus, both at admission and during hospitalization, as well as the need for cerebrospinal fluid diversion procedures such as shunt placement. Data on the modality used for repeat vascular imaging, namely, DSA, CTA, or MRA, were also recorded. Patients were stratified into two groups based on the modified Rankin Scale (mRS) score at discharge: the good outcome group (mRS 0-2) and the poor outcome group (mRS 3-6). Statistical analysis was conducted using the EZR software version 2.9 (Saitama Medical Center, Jichi Medical University, Saitama, Japan), and comparisons between groups were performed using the Mann-Whitney U test or Fisher's exact test, with a significance threshold of p<0.05.

## Results

Among the 26 patients included in this study (Table 1), 16 achieved good functional outcomes (mRS 0-2) at discharge, while 10 experienced poor outcomes (mRS 3-6). Patients with good outcomes exhibited significantly higher GCS scores at admission, with a mean score of 15 compared to 13 in the poor outcome group (p=0.008). Loss of consciousness at presentation was more frequently observed in the poor outcome group (40% vs. 6.3%; p=0.055), although this did not reach statistical significance (Table 2).

**Table 1:** Baseline demographic and clinical characteristics of patients with unknown SAH This table summarizes patient characteristics at the time of admission, including age, sex, initial GCS score, grading scores (WFNS, Fisher, and H&K), presence of perimesencephalic bleeding, loss of consciousness, and hydrocephalus. SAH: subarachnoid hemorrhage; GCS: Glasgow Coma Scale; WFNS: World Federation of Neurosurgical Societies; H&K: Hunt and Kosnik

	n=26
Age (years) (mean)	65.3
Sex (male)	14 (53.8%)
GCS on admission	12.8
WFNS, Fisher, H&K	1.85, 2.5, 1.62
Perimesencephalic SAH	10 (38.5%)
Loss of consciousness	5 (19.2%)
Hydrocephalus on admission	6 (23.1%)

**Table 2:** Comparison of consciousness level and demographics by outcome Comparison of age, sex, initial GCS score, and incidence of loss of consciousness between good outcome (mRS 0-2) and poor outcome (mRS 3-6) groups. GCS was significantly higher in the good outcome group. mRS: modified Rankin Scale; GCS: Glasgow Coma Scale

	Good (mRS 0-2) n=16	Poor (mRS 3-6) n=10	P-value
Age (years) (mean)	67.1±13.3	71.1±10.6	0.224
Sex (male)	9 (56.3%)	5 (50%)	1
GCS on admission	15 (14.5-15)	13 (7.5-14.5)	0.008
Loss of consciousness	1 (6.3%)	4 (40%)	0.055

The hemorrhage pattern was classified as focal or diffuse based on initial imaging. Focal hemorrhage was defined as localized bleeding confined to the perimesencephalic cisterns or a limited subarachnoid area, without extension into the Sylvian fissures or ventricles. Diffuse hemorrhage was defined as blood widely distributed across multiple cisterns, fissures, or ventricles. Focal hemorrhage distribution was significantly more common among patients with good outcomes (81.3%) compared to those with poor outcomes (40%) (p=0.046). Lower WFNS (median 1 vs. 2; p=0.007), Fisher group (median 2 vs. 3; p=0.044), and H&K grades (median 1 vs. 2; p=0.012) were also associated with good outcomes (Table 3). Hydrocephalus at admission (6.3% vs. 50%; p=0.018) and during hospitalization (6.3% vs. 40%; p=0.034) was more common in the poor outcome group. Shunt placement was required in 20% of the poor outcome group and in none of the good outcome group (p=0.046) (Table 4).

**Table 3:** Hemorrhage pattern and clinical grading scores by outcome Distribution pattern of hemorrhage (focal vs. diffuse) and initial grading scores including WFNS, Fisher, and H&K grades. Focal hemorrhage and lower severity scores were significantly associated with good outcomes. mRS: modified Rankin Scale; SAH: subarachnoid hemorrhage; WFNS: World Federation of Neurosurgical Societies; H&K: Hunt and Kosnik

	Good (mRS 0-2) n=16	Poor (mRS 3-6) n=10	P-value
Perimesencephalic SAH	6 (37.5%)	4 (40%)	1
Focal hemorrhage	13 (81.3%)	4 (40%)	0.046
WFNS (median)	1.3 (1-1)	2.5 (1-4)	0.007
Fisher (median)	2.3 (2-3)	2.7 (2-3)	0.044
H&K (median)	1.3 (1-1)	2.0 (1-3)	0.012

**Table 4:** Complications (hydrocephalus, shunt, and spasm) by clinical outcome group Rates of hydrocephalus at admission and during hospitalization, as well as the proportion of patients requiring CSF shunt placement. These complications were more frequent in the poor outcome group. mRS: modified Rankin Scale; CSF: cerebrospinal fluid

	Good (mRS 0-2) n=16	Poor (mRS 3-6) n=10	P-value
Hydrocephalus on admission	1 (6.3%)	5 (50%)	0.018
Hydrocephalus during hospitalization	1 (6.3%)	4 (40%)	0.034
Shunt drainage	0 (0%)	2 (20%)	0.046
Spasm (symptomatic)	3 (18.85%)	2 (20%)	0.697

Repeat angiography using DSA was performed more frequently in the good outcome group (53.3%) compared to the poor outcome group (10%) (p=0.022), although no new vascular lesions were identified in any patients (Table 5).

**Table 5:** Re-angiography modality and association with clinical outcome Comparison of re-angiography modalities (DSA, CTA, MRA) between good and poor outcome groups. DSA was more frequently used in patients with good outcomes and was the only modality significantly associated with outcome. The continuous variable was analyzed using the Mann-Whitney U test. Categorical variables were analyzed using Fisher's exact test. A p-value of <0.05 was considered statistically significant. mRS: modified Rankin Scale; DSA: digital subtraction angiography; CTA: computed tomography angiography; MRA: magnetic resonance angiography

	Good (mRS 0-2) n=15	Poor (mRS 3-6) n=10	P-value
Number of angiographies performed	2.9 (2-4)	2.45 (1.5-4)	0.177
Proportion of patients who underwent more than two angiographies	12 (80%)	8 (80%)	1
Method of the first angiography on admission			
DSA	8 (53.3%)	9 (90%)	0.123
CTA	4 (26.7%)	1 (10%)	
MRA	4 (26.7%)	0 (0%)	
Timing of the second angiography	4 (26.7%)	6 (60%)	0.67
Method of the second angiography			
DSA	8 (53.3%)	5 (50%)	0.022
CTA	6 (40%)	1 (10%)	
MRA	2 (13.3%)	0 (0%)	

## Discussion

Timing of initial vascular evaluation

Early vascular imaging remains the cornerstone of diagnostic assessment in patients with SAH. DSA is widely considered the gold standard due to its superior spatial resolution and sensitivity for detecting small or complex aneurysms [1,4,5]. However, its initial diagnostic yield may be compromised by clot formation, vasospasm, or technical error. Kikuchi et al. highlighted that even when DSA is promptly performed, a significant number of cases remain angiographically negative due to interpretive or procedural limitations [4].

Recent recommendations suggest performing DSA within 24-48 hours of ictus to maximize diagnostic accuracy, particularly in patients with non-perimesencephalic bleeding patterns [2]. Although timing alone does not guarantee success, early imaging offers a valuable baseline and allows for more informed decisions regarding the need for follow-up angiography.

In our findings, while initial DSA was performed in all patients within 24 hours of hospital admission, its yield remained zero, echoing concerns from previous literature that a single negative angiogram does not exclude vascular pathology [1,7].

Optimal timing for re-angiography

The decision of when to repeat angiographic imaging after an initial negative DSA remains a point of debate. Literature suggests a biphasic approach: early re-angiography (within 7-14 days) to detect vasospasm-masked aneurysms and delayed imaging (4-6 weeks later) for evolving lesions like dissecting aneurysms or de novo fistulas [1,6]. Dalyai et al. reported that short-term (one-week) and long-term (six-week) follow-up angiographies revealed vascular abnormalities in 12.5% of non-perimesencephalic SAH patients [7].

Ohshima et al. documented cases where dural arteriovenous fistulas (dAVFs) were identified months after initial negative imaging, demonstrating that delayed evaluations may uncover evolving pathologies [6]. Additionally, Vogetseder et al. described an aneurysm only visible four months after SAH onset, reinforcing the need for long-term surveillance in select patients [8].

Despite a lack of re-detected lesions in our cohort, the literature supports repeat imaging in high-risk presentations, particularly diffuse or non-perimesencephalic hemorrhages [2,9].

Preferred method and frequency of re-angiography

Although non-invasive modalities like CTA and MRA are frequently employed for follow-up due to their safety profile, they may lack the resolution needed to detect subtle vascular anomalies. Kikuchi et al. found that in cases where CTA and MRA failed to identify lesions, only repeat DSA uncovered the underlying cause [4]. Similarly, Mehdorn et al. emphasized the diagnostic yield of second and even third DSAs, with aneurysms detected in a considerable proportion of initially negative patients [1].

Larson and Brinjikji proposed a bleeding pattern-based stratification: patients with perimesencephalic hemorrhage may require no further imaging after negative DSA, whereas those with diffuse hemorrhage should undergo repeated DSA [2]. Meta-analyses support this approach, suggesting limited yield in perimesencephalic cases but substantial benefit in more extensive SAH [7].

Nevertheless, frequent invasive imaging carries procedural risks and patient burden. In our cohort, repeat angiography was performed in 22 out of 26 patients (84.6%), with DSA used in 13 cases and CTA in nine cases. DSA was generally preferred when detailed vascular assessment was required, while CTA was selected in clinically stable patients or when a non-invasive approach was considered more appropriate. A balanced strategy involves tailored re-imaging protocols based on hemorrhage pattern, initial grading scores, and clinical evolution, as emphasized by recent multicenter reviews [8,10].

Our study uniquely highlights that early radiological and clinical features, specifically focal hemorrhage pattern, high GCS scores at admission, and absence of hydrocephalus, are more predictive of short-term outcomes than the findings of repeat angiography. These factors can potentially serve as simple yet powerful tools for early prognostic assessment in patients with unknown SAH. Notably, repeat angiography using DSA was more frequently performed in patients with good outcomes. This association has not been clearly described in previous studies. Although no new vascular lesions were identified, this trend may reflect the clinical stability of these patients, making invasive imaging more feasible, or it may indicate a difference in physician decision-making.

Based on our findings and previous literature, we propose a pragmatic imaging approach: in patients with focal hemorrhage patterns and favorable neurological status, a single negative DSA may be sufficient. In contrast, those presenting with diffuse hemorrhage or clinical instability may benefit from repeated angiographic evaluation, including DSA or CTA at different time points. While a formal algorithm was beyond the scope of this retrospective study, our data suggest that imaging decisions should be individualized based on hemorrhage distribution and clinical course.

Limitations

This study has several limitations. First, it was a single-center, retrospective analysis with a relatively small sample size, which may limit the generalizability of the findings. Second, the assessment of outcomes was restricted to the time of hospital discharge, and long-term functional status was not evaluated. Third, although repeat angiography was analyzed, its indication and timing were not standardized and may have been influenced by clinician preference or patient condition. Further prospective multicenter studies are needed to validate these findings and to establish standardized imaging protocols for angiogram-negative SAH.

## Conclusions

Unknown SAH typically has a favorable prognosis, particularly in patients with focal hemorrhage and stable neurological status. However, initial negative angiography cannot rule out a vascular cause. While repeat DSA did not reveal any new lesions in our cohort, its more frequent use in patients with good outcomes suggests it may play a supportive role in management. A tailored approach, prioritizing DSA in high-risk patients and limiting follow-up in low-risk cases, offers a pragmatic balance between diagnostic accuracy and procedural burden. Further prospective studies are needed to develop standardized, risk-stratified imaging protocols to improve patient outcomes and resource utilization.
